# The pulmonary resistance‐compliance relationship: Real or mathematical artifact?

**DOI:** 10.14814/phy2.70355

**Published:** 2025-05-05

**Authors:** Sara Hungerford, Navin Kapur, Stuart Rich, Daniel Burkhoff

**Affiliations:** ^1^ The CardioVascular Center Tufts Medical Center Boston Massachusetts USA; ^2^ Faculty of Health and Medicine University of Sydney Sydney Sydney NSW Australia; ^3^ Department of Cardiology Royal North Shore Hospital Sydney Sydney NSW Australia; ^4^ Northwestern University Chicago Illinois USA; ^5^ Cardiovascular Research Foundation New York New York USA

**Keywords:** pulmonary arterial compliance, pulmonary hypertension, pulmonary vascular resistance

## Abstract

Pulmonary vascular resistance (PVR) and arterial compliance (PAC) have been reported to share an inverse hyperbolic relationship, with their product—the pulmonary vascular time constant (τ)‐remaining more or less constant across disease states. However, PAC is often estimated as stroke volume (SV) divided by pulse pressure (PAC_clinical_ = SV/PP), introducing potential mathematical coupling with PVR, which is dependent on SV. This inherent relationship may artificially produce hyperbolic correlations. We conducted joint density analysis (JDA) and hemodynamic simulations using data from patients with pulmonary hypertension due to left‐sided disease. PAC_clinical_ was calculated as SV/PAPP, and PVR as (PA_M_ ‐ PCWP)/CO. Log‐transformed values were analyzed to assess the PVR‐PAC_clinical_ relationship. JDA demonstrated a strong inverse correlation between PVR and PAC_clinical_ (Spearman's *R* = −0.88, CI −0.92 to −0.82), increasing to −0.92 with bootstrapping. Hemodynamic simulations confirmed a first‐order hyperbolic decay (PAC = τ_avg/_PVR; *r* ^2^ = 0.86, *p* < 0.001). The relationship shifted downward with increasing PCWP. Our findings replicate the reported PVR‐PAC_clinical_ relationship, highlighting its mathematical dependence. Further studies using more accurate PAC estimation methods are needed to determine whether this association is truly physiological or an artifact of calculation.

## 1 INTRODUCTION

Measurement of pulmonary arterial compliance (PAC)—the pulsatile component of vascular afterload—is of importance for the determination of right ventricular (RV)‐PA coupling in health and disease. Historically, PAC and pulmonary vascular resistance (PVR)—the steady component of vascular afterload—are considered to be physiologically linked through an inverse hyperbolic relationship, with their product (the pulmonary vascular time constant [τ]), thought to remain relatively constant across disease states (i.e., any rise in PVR is followed by an inverse reduction in PAC, and vice versa) (Lankhaar et al., 2006). However, more recent studies suggest that τ may be influenced by changes in pulmonary capillary wedge pressure (PCWP) (Grignola et al., 2019; Metkus et al., 2016; Tedford et al., 2012). A critical concern emerges from the way PVR and PAC are routinely quantified, as we discuss further below.

From a physiological standpoint, PAC is most accurately defined as the increase in PA blood volume (∆V) that produces a unit increase in transmural pressure (∆P). Whereas ∆P can be quantified accurately through high fidelity measurements of PA pulse pressure (PA_PP_), quantification of ∆V is not possible since a significant fraction of the stroke volume (SV) is discharged out of the PA system during ejection. Disregarding this fundamental limitation, PAC has come to be estimated empirically in many realms of clinical research as the ratio of SV to PA_PP_ (PAC_clinical_) (Chemla et al., 1998). This is despite the fact that more precise methods of quantification (i.e., diastolic decay exponential fitting and area under the curve [AUC] method which, in both cases, need to account for the fact that PA pressure decreases toward the pulmonary venous pressure and not to 0 mmHg) have been described. (Liu et al., 1986) Accordingly, when PVR is calculated as the ratio of PA_M_ to mean flow, and PAC_clinical_ the ratio of SV to PP, for *τ* it the holds that:

$$\tau =R\;X\;C=\frac{\mathrm{PAm}-\text{PCWP}}{\mathrm{SV}/T}X\;\frac{\mathrm{SV}}{\mathrm{PP}}=T\frac{\mathrm{PAm}-\text{PCWP}}{\mathrm{PP}}$$

With *T* the heart period and PCWP the pulmonary capillary wedge pressure (Lankhaar et al., 2006).

In context of the above, it is relevant to explore the foundation of the reported link between PVR and PAC because they share the value of SV in their determination; in the numerator in the case of PAC_clinical_, and in the denominator in the case of PVR. In other words, plotting a parameter that is dependent on SV (i.e., PVR) versus another that is dependent on 1/SV (i.e., PAC_clinical_) is inherently prone to yield a hyperbolic relationship.

## 2 METHODS

To test our hypothesis, we performed: (1) joint density analysis (JDA), and (2) a hemodynamic simulation based on previously published data from a cohort of patients with PH from left‐sided disease (Burkhoff et al., 2021). Study data are available upon request. All analyses were performed using standard functions in Microsoft Excel and R (version 4.4.3), without custom code or algorithms.

To perform JDA, we fit a gamma‐distributed generalized linear model (GLM) with a log link function in R. Model residuals and fit diagnostics were visually inspected. Spearman's correlation coefficient (ρ) and bootstrapped confidence intervals (CI) (1000 iterations) were used to quantify and validate the observed correlation between PVR and PAC_clinical_.

**FIGURE 1:**
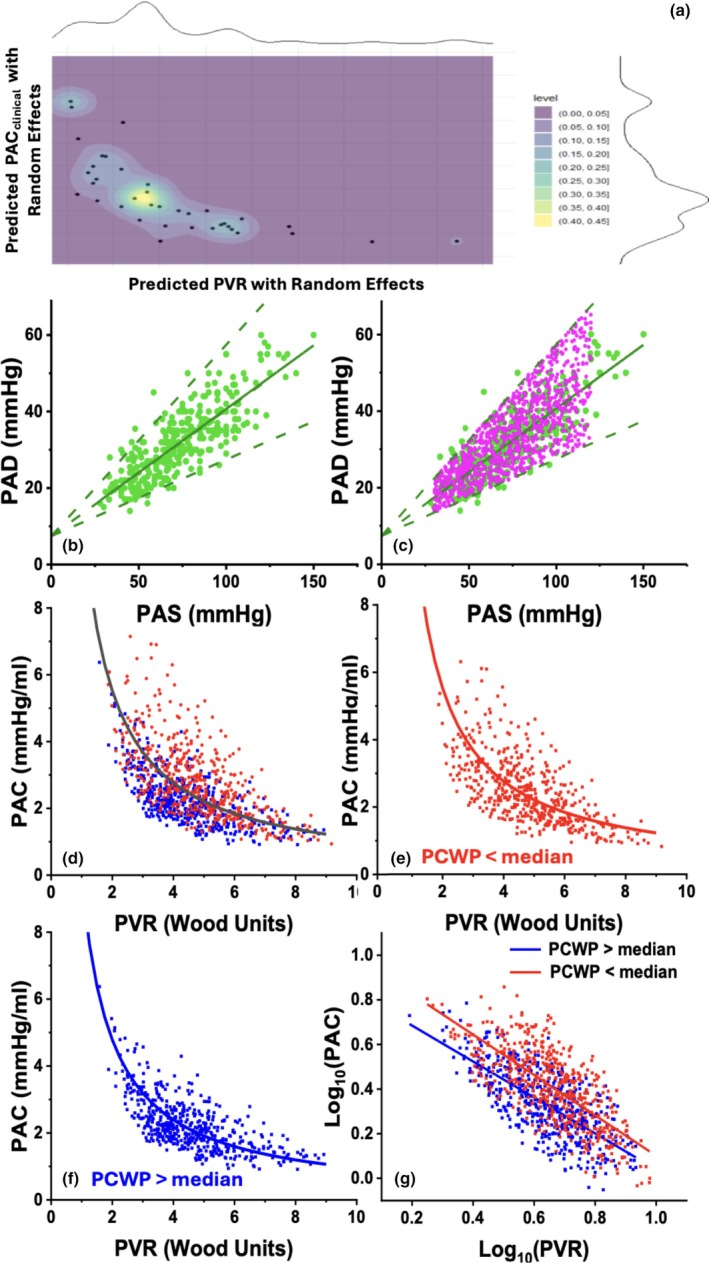
(a) JDA of PVR and PAC_clinical_ from a cohort of patients with PH from left‐sided disease was performed to understand how the two variables were related mathematically. Color indicates the density (or frequency) of data points for PVR and PAC_clinical_. A mixed model was run, fit with a gamma distribution and log link. The plotted model predicted PAC_clinical_ (y‐axis) against model predicted PVR (x‐axis). This gave a Spearman's R of −0.88 (CI −0.92 to −0.82, via Fischer's Z transform). The data was bootstrapped so that each subject was only sampled once and resampled 10,000 times, producing a Spearman's R of −0.92 (CI −0.93 to −0.91, bias corrected). With raw bootstrapping the Spearman's R increased to −0.93 (CI −1.00 to −0.66, bias corrected). Overall, JDA demonstrated very high mathematical correlation between PVR and PAC_clinical_. (b) Scatterplot of the PA_S_ (x‐axis, mmHg) and PA_D_ (y‐axis, mmHg) relationship in the cohort showing strong correlation between the two (*r* ^2^ 0.80; *p* < 0.001). The average slope of the relationship was 0.33 (range 0.2–0.5). (c) Scatterplot of 1000 random simulation values of PA_S_ (x‐axis, mmHg) and PA_D_ (y‐axis, mmHg) again showing close correlation between the two (*r* ^2^ 0.79; *p* < 0.001). (d) Scatterplot of 1000 random simulation values of PVR (x‐axis; WU) and PAC (y‐axis; mL/mmHg) showing (black line) an inverse hyperbolic relationship (*r* ^2^ 0.86; *p* < 0.001). PAC was predicted by *τ*_*avg*_ */PVR* where *τ* is the group average *τ* determined by PVR*PAC from each individual pair. (e, f) Scatterplot of the effect of PVR (x‐axis; WU) and PAC (y‐axis, mL/mmHg) relationship by change in PCWP above (blue) or below median (red; 14.2 mmHg). The relationship was shift downward in a parallel manner for those with a PCWP > median. (g) Generalized linear regression relationship of Log_10_ PVR (x‐axis) and Log_10_ PAC (y‐axis) by change in PCWP above (blue) or below median (red; 14.2 mmHg). With increased PCWP the curve shifted downward in a parallel manner. JDA, joint density analysis; PA, pulmonary artery; PAC, pulmonary arterial compliance; PCWP, post‐capillary wedge pressure; PVR, pulmonary vascular resistance; WU, Wood Units.

Secondly, we performed a physiologically constrained hemodynamic stimulation with the following steps: (1) determined the relationship between PA systolic (PA_S_) and PA diastolic (PA_D_), Figure 1b; (2) generated 1000 random PA systolic (PA_S_) values between 30–120 mmHg; (3) calculated PA diastolic (PA_D_) using a physiologically plausible linear regression (*PA*_*S*_ *x* [*random value between 0.2 and 0.5*] *+ 7.4*); (4) computed PA_M_ (*PA*_*D*_ + [*PA*_*S*_ *– PA*_*D*_]*/3*); (5) estimated PCWP by subtracting a random value (0–20 mmHg) from PA_D_; (6) generated CO (3.5–6.5 L/min) and HR (60–85bom) to derive SV (*CO/HR*); (7) calculated PVR, PAC_clinical_ and τ; (8) log‐transformed the data to assess linearity and PCWP‐related shifts. All parameters were randomly sampled from uniform distributions within physiological ranges. A total of 1000 replicates were generated, and a fixed random seed was used to ensure reproducibility.

## 3 RESULTS

JDA is shown in Figure 1a below. The gamma‐distributed GLM with a log link showed a strong inverse PVR‐PAC_clinical_ relationship with good model fit. Spearman's ρ was −0.88 (95% CI –0.92 to −0.82), improving to −0.92 (95% CI –0.93 to −0.91) after bootstrapping, indicating excellent correlation and precision.

Hemodynamic simulation results are shown in Figure 1b–f. A strong linear relationship was observed between PA_S_ and PA_D_ (*PA*_*D*_ *= 7.4 + 0.33·PA*_*S*_, *r* ^2^ = 0.80, *p* < 0.001; slope range 0.2–0.5; Figure 1b,c). Simulated τ values averaged 0.59 ± 0.22 s (range 0.24–1.63 s), consistent with prior reports. Modeled data showed an inverse PVR–PAC_clinical_ relationship characterized by a first‐order hyperbolic decay function (*PAC = τ*_*avg*_ */PVR*, *r* ^2^ = 0.86, *p* < 0.001; Figure 1d). Stratification by PCWP (median 14.2 mmHg) revealed a parallel downward shift in the log‐transformed PVR–PAC_clinical_ relationship (Figure 1e,f). Compared with data from points associated with lower PCWP, the log_10_‐transformed relationship was shifted downward in a nearly parallel manner: *log*_*10*_(*PAC*) *= 0.71–0.83∙log*_*10*_(*PVR*) (*r* ^2^ = 0.68) above median versus *log*_*10*_(*PAC*) *= 0.79–0.89∙log*_*10*_(*PVR*) (*r* ^2^ = 0.65) for PCWP below median. ANCOVA confirmed a significant interaction between PCWP group and log_10_(PVR) (*p* = 0.008). A significant intercept shift (*p* < 0.001) confirmed lower PAC_clinical_ values in the high‐PCWP group.

## 4 DISCUSSION

By performing JDA and physiologically constrained hemodynamic simulations, we reproduced with high fidelity the inverse hyperbolic relationship between PVR and PAC clinically reported in prior studies (Lankhaar et al., 2006), along with the modifying effect of PCWP (Grignola et al., 2019; Metkus et al., 2016; Tedford et al., 2012) Taken together, our findings suggest this relationship may not exclusively reflect a fundamental property of the pulmonary vasculature, but rather the result of plotting mathematically coupled variables with a predictable shift based on PCWP.

Our findings contradict prior work using semi‐logarithmic modeling, which propose a physiologic basis for RC‐time independent of SV. In a prior study by *Ghio* et al., the authors reported that PVR‐PAC_clinical_ consistency reflected the influence of SV on pulsatile pressure generation rather than its absolute value–underscoring the interplay between flow, pressure, and compliance (Ghio et al., 2017). More accurate PAC estimation methods–such as exponential fitting of diastolic pressure decay or AUC analysis–may better account for pressure decay toward pulmonary venous pressure and offer more physiologically sound alternatives to SV/PP‐based estimation (Liu et al., 1986). Future studies using cardiac MRI or 4D echocardiography may enhance ΔV and PAC quantification by incorporating left atrial reservoir function and pulmonary venous return, potentially resolving discrepancies between decay, AUC, and SV/PP methods.

Our study has limitations. PA_M_ was calculated assuming a fixed PA pressure contour, potentially reducing its independence from PA_S_ and PA_D_ and introducing mathematical coupling in the estimation of τ. In reality, τ varies with diastolic pressure decay, particularly in PH due to left‐sided heart disease, where elevated PCWP alters afterload. While the average τ (~0.59 s) was consistent with prior studies, the broader range observed (0.24–1.63 s) may reflect both physiologic extremes and/or artifacts from implausible parameter combinations introduced by uniform random sampling. This underscores the importance of interpreting τ within a clinical context and supports the use of physiologically constrained models to avoid overinterpreting values unlikely to arise in real‐world patients. Finally, we did not isolate HR as an independent variable in this analysis. Future work to understand whether HR exerts a measurable influence on τ—particularly at more extreme ranges—is required.

## 5 CONCLUSIONS

Our findings suggest the inverse, hyperbolic PVR–PAC_clinical_ relationship may arise from the mathematical coupling of SV rather than a true physiological property. Further studies using more precise PAC methods—particularly in patients with left‐sided PH—are required to determine whether this relationship remains absolute across all PH subtypes or is simply a mathematical artifact.

## FUNDING INFORMATION

None.

## ETHICS STATEMENT

The research was prospectively reviewed and approved by the Northwestern University Feinberg School of Medicine ethics committee (NCT03541603).
